# Diffuse Unilateral Subacute Neuroretinitis Caused by *Ancylostoma* Hookworm

**DOI:** 10.3201/eid2302.142064

**Published:** 2017-02

**Authors:** Sven Poppert, Martin Heideking, Hansjürgen Agostini, Moritz Fritzenwanker, Nicole Wüppenhorst, Birgit Muntau, Philipp Henneke, Winfried Kern, Jürgen Krücken, Bernd Junker, Markus Hufnagel

**Affiliations:** University Medical Center, Hamburg-Eppendorf, Hamburg (S. Poppert);; Justus-Liebig-University Giessen Institute for Medical Microbiology, Giessen, Germany (S. Poppert, M. Fritzenwanker);; Bernhard Nocht Institute for Tropical Medicine, Hamburg, Germany (S. Poppert, B. Muntau);; University Hospital Tübingen Children’s Hospital, Tübingen, Germany (M. Heideking);; University Medical Center Freiburg Center of Pediatrics and Adolescent Medicine, Freiburg, Germany (M. Heideking, P. Henneke, M. Hufnagel);; University Medical Center Freiburg Eye Center, Freiburg (H. Agostini, B. Junker);; Institute for Hygiene and Environment, Hamburg (N. Wüppenhorst);; University Medical Center Freiburg Department of Gastroenterology, Hepatology, Endocrinology, and Infectious Diseases, Freiburg (W. Kern);; Institute for Parasitology and Tropical Veterinary Medicine, Freie Universität Berlin, Berlin, Germany (J. Krücken);; Hannover Medical School, Eye Clinic, Hannover, Germany (B. Junker)

**Keywords:** diffuse unilateral subacute neuroretinitis, DUSN, ocular, infectious disease, nematodes, hookworm, Ancylostoma ceylanicum, Baylisascaris procyonis, Toxocara canis, parasites, zoonoses

## Abstract

Diffuse unilateral subacute neuroretinitis is an ocular infectious disease caused by several distinct nematodes. Definite identification of the involved nematodes is rarely achieved. We report on the molecular-based genetic identification of an *Ancylostoma ceylanicum* hookworm implicated in a case of diffuse unilateral subacute neuroretinitis in a child.

Diffuse unilateral subacute neuroretinitis (DUSN) is an ocular infectious disease caused by migrating larvae of nematodes. Patients typically have vitritis, papillitis, and gray-white retinal lesions (*1**,**2*). DUSN primarily occurs in the United States, the Caribbean, and South America, although several cases also have been reported in Europe, Africa, India, and China (*2*). Several nematodes can induce DUSN, in particular *Ancylostoma* spp.*, Baylisascaris procyonis*, and *Toxocara canis* (*3*), but the actual cause remains unknown. Because the nematodes are only rarely surgically extracted from the eye, a definite identification is hardly ever achieved (*3**,**4*). Noninvasive laser therapy is the treatment of choice for DUSN because it leads to the death of the nematode, thereby stopping the inflammatory process (*1*). Anthelminthic therapy with albendazole also has been described as successful, albeit primarily in cases where a worm cannot be visualized in the patient’s eye (*5*). Left untreated, DUSN can progress toward optic nerve atrophy and permanent vision loss.

In their larval form, hookworms infect their hosts by penetrating intact skin. The larvae circulate through the blood to the heart and then reach the lungs, before being coughed up and swallowed, thus entering the gastrointestinal tract. In the intestines, the larvae develop into adult worms and start to reproduce. This leads to the fecal shedding of eggs into the environment. *A. ceylanicum* is a zoonotic hookworm predominantly found in dogs and cats in Southeast Asia, India, and Australia (*6*). It is the only animal hookworm species known to cause patent intestinal infections in humans (*6*).

We report on a 10-year-old boy born in Columbia who had been living with his foster parents in Germany for the previous 6 years. He had acute loss of vision in his right eye. Ophthalmoscopy revealed retinal vasculitis, exudative retinal detachment, and proliferative vitreoretinopathy. Because of the retinal detachment, we performed a vitrectomy, during which a white worm of ≈10 mm in length was observed moving in the subretinal space (Figure). During surgical removal, the worm was completely destroyed. 

**Figure:**
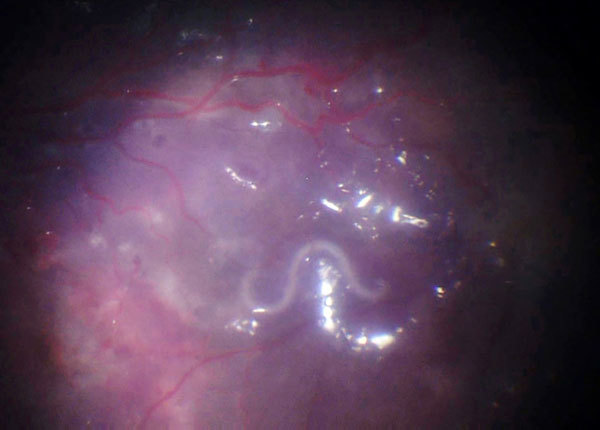
Intraoperative fundus image depicting a migrating hookworm (*Ancylostoma ceylanicum*) ≈10 mm in length in the subretinal space of the eye of 10-year-old patient in Germany.

We isolated DNA from the intraoperative rinsing fluid and applied previously described PCR assays to target the mitochondrial 12S rRNA, Cox1, and intergenic transcribed spacer 1 and 2 of various nematodes (*7**–**9*). We then sequenced the PCR products obtained. Phylogenetic analysis with the intergenic transcribed spacer 1 and 2 sequences (GenBank accession no. KM066110.1) identified the worm as *A. ceylanicum* (Technical Appendix). A blastn search (https://blast.ncbi.nlm.nih.gov) with the Cox1 sequence (GenBank accession no. KM066109.1) showed 99% and 87% identity with *A. ceylanicum* and *A. caninum*, respectively. The 12Sr RNA sequence (GenBank accession no. KM066111.1) for *A. ceylanicum* revealed 94% and 92% identity to *A. caninum* and *A. duodenale,* respectively.

Additional diagnostic results included negative serum antibody tests for the parasites *Fasciola hepatica*, *Strongyloides* spp., *Trichinella* spp., and *Taenia solium*; negative blood samples for filariasis; and 3 negative stool samples for intestinal helminths. An ELISA result for serum antibodies to the helminth *Toxocara canis* (DRG, Marburg, Germany) was weakly positive, whereas the confirmatory immunoblot test result (Lobio Diagnostics, Lyon, France) was negative.

In addition to performing a vitrectomy, retinotomy, and implantation of silicon oil, we started the patient on antiinflammatory therapy with oral prednisone. Referring to the treatment recommendation of a case series (*5*) and ensuring that no additional worm could survive, we administered a 30-day course of anthelminthic therapy with albendazole, even though no signs of additional organ manifestation or blood eosinophilia were observed. Unfortunately, a permanent loss of visual acuity to 0.05 could not be averted.

DUSN is an inflammatory eye disease caused by migrating nematode larvae. Because of the surgical intervention necessary in the case we describe, we were able to amplify and determine DNA sequences of the hookworm *A. ceylanicum.* The finding of a hookworm in DUSN seems plausible because *Ancylostoma* spp. nematodes have been repeatedly proposed as an etiologic agent in DUSN (*1**,**3**,**4*).

The source and time of infection in our patient remains unclear. After the patient’s adoption and his move from Columbia to Germany at 4 years of age, the patient and his adoptive family spent vacations in Spain but never traveled outside Europe. We do not know whether the infection was acquired in Columbia or Spain and subsequently survived (e.g., in a hypobiotic state) or whether the infection was acquired in Germany. Although the definitive source and time of infection cannot be confirmed, molecular methods nevertheless unquestionably identified the species *A. ceylanicum.*

In this case, we obtained a positive ELISA result for *Toxocara canis*, another helminth implicated in DUSN (*3*). Because the confirmatory immunoblot test was negative, we assume the ELISA result to most likely have been caused by unspecific cross-reactions. In the past, tests based on serologic testing alone might have falsely attributed nematodes to DUSN. In future cases of exudative retinal detachment caused by DUSN, intraoperative material should be used for molecular studies to identify the responsible nematode.

Technical Appendix Phylogram of intergenic transcribed spacer 1 and 2 sequences from *Ancylostoma* and *Uncinaria* spp.
