# Supplementary material for: Diffuse Unilateral Subacute Neuroretinitis Caused by Ancylostoma Hookworm
Source: Emerg Infect Dis. 2017 Feb;23(2):343–4. doi: 10.3201/eid2302.142064 (PMC5324813; doi:10.3201/eid2302.142064)
Supplement: Technical Appendix — Phylogram of intergenic transcribed spacer 1 and 2 sequences from Ancylostoma and Uncinaria spp. [file 14-2064-Techapp-s1.pdf]

Article DOI: http://dx.doi.org/10.3201/eid2302.142064

Diffuse Unilateral Subacute Neuroretinitis Caused by *Ancylostoma* Hookworm

Technical Appendix

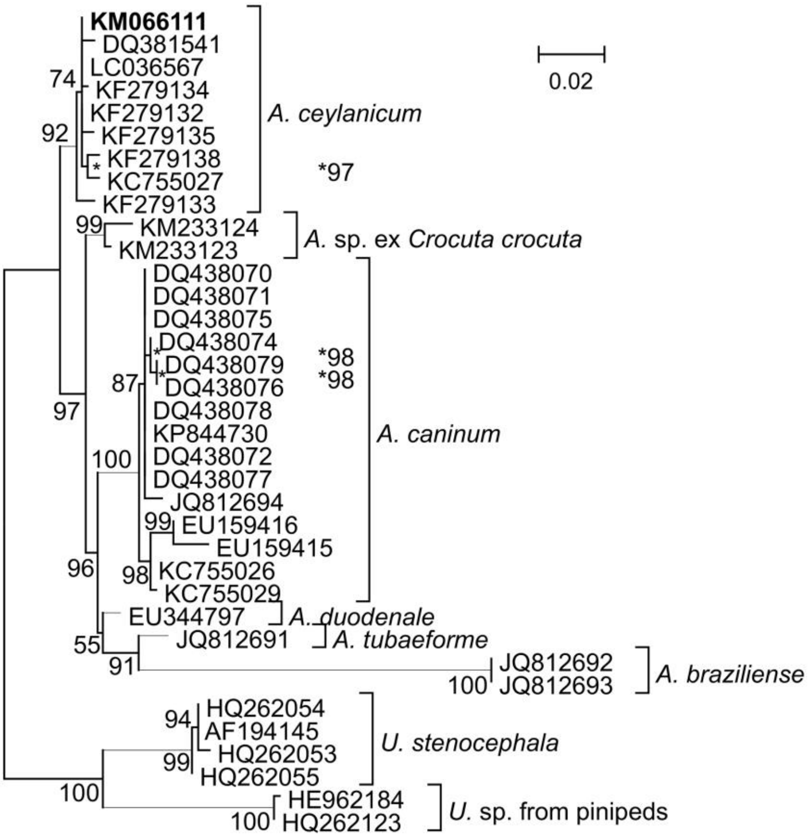

Technical Appendix Figure. All intergenic transcribed spacer 1 and 2 sequences (GenBank accession no. KM066110.1) from *Ancylostoma* spp. and a few *Uncinaria* spp. as outgroup were aligned separately by using MAFFT (http://mafft.cbrc.jp/alignment/software) with the Q-INS-I to consider RNA secondary structure. The phylogram was calculated by using PhyML3.1 (*1*) and applying a GTR+I+F+G model with 25 substitution rate categories based on the concatenated alignments. Branch support was obtained by using a Bayesian transformation of the approximate-likelihood-ratio test. Sequences are represented by GenBank accession numbers, and the specimen found in the present study is shown in bold type.

Reference

1. Guindon S, Dufayard JF, Lefort V, Anisimova M, Hordijk W, Gascuel O. New algorithms and methods to estimate maximum-likelihood phylogenies: assessing the performance of PhyML 3.0. Syst Biol. 2010;59:307–21. PubMed http://dx.doi.org/10.1093/sysbio/syq010
